# RBM3 suppresses stemness remodeling of prostate cancer in bone microenvironment by modulating N6-methyladenosine on CTNNB1 mRNA

**DOI:** 10.1038/s41419-023-05627-0

**Published:** 2023-02-07

**Authors:** Shouyi Zhang, Chengcheng Lv, Yichen Niu, Changqi Li, Xiuming Li, Yu Shang, Yunchao Zhang, Yue Zhang, Yong Zhang, Yu Zeng

**Affiliations:** 1 Department of Urology, the Cancer Hospital of Dalian University of Technology & Liaoning Cancer Hospital, Shenyang, Liaoning 110042 China; 2 Department of Laboratory Medicine, the First Affiliated Hospital of China Medical University, Shenyang, Liaoning 110001 China; 3 Department of Urology, the Affiliated Hospital of Chengde Medical University, Chengde, Hebei 067000 China; 4 Department of Oncology, the Second Hospital of Dalian Medical University, Dalian, Liaoning 116000 China; 5 Department of Pathology, the Cancer Hospital of Dalian University of Technology & Liaoning Cancer Hospital, Shenyang, Liaoning 110042 China

**Keywords:** Cancer microenvironment, RNA modification

## Abstract

Bone metastasis is the most happened metastatic event in prostate cancer (PCa) and needs a large effort in treatment. When PCa metastasizes to the bone, the new microenvironment can induce the epigenome reprogramming and stemness remodeling of cancer cells, thereby increasing the adaptability of cancer cells to the bone microenvironment, and this even leads to the occurrence of secondary tumor metastasis. Our group has previously found that RNA binding motif 3 (RBM3) affects the stem cell-like properties of PCa by interfering with alternative splicing of CD44. However, whether RBM3, as a stress-response protein, can resist microenvironmental remodeling of PCa particularly in bone metastasis remains unknown. By co-culturing PCa cells with osteoblasts to mimic PCa bone metastases, we found that RBM3 upregulates the N6-methyladenosine (m^6^A) methylation on the mRNA of catenin beta 1 (CTNNB1) in a manner dependent on methyltransferase 3 (METTL3), an N6-adenosine-methyltransferase complex catalytic subunit. Consequently, this modification results in a decreased stability of CTNNB1 mRNA and a followed inactivation of Wnt signaling, which ultimately inhibits the stemness remodeling of PCa cells by osteoblasts. Thus, the present study may extend our understanding of the inhibitory role of RBM3 on particularly bone metastasis of PCa.

## Introduction

Prostate cancer (PCa) is one of the most frequently diagnosed cancers and is a leading cause of death from cancers in men worldwide. The majority of metastatic PCa will eventually progress to castration resistant PCa, which is the lethal stage of the disease. Notably, metastasizing to the bone happens almost without exception in PCa. Generally, bone metastases develop as follows: [1] colonization of circulating disseminated tumor cells (CTCs); [2] CTCs lie dormant in the bone microenvironment; [3] reactivation and development of new cell colonies; and [4] remodeling of the bone microenvironment by tumor cells. It has been shown that Wnt signaling plays an important role in these processes [1, 2]. The bone microenvironment is not only suitable for the survival of CTCs, but also endows the tumor cells with stem-like properties, which help cancer cells develop drug resistance and contribute to secondary metastasis from bone [3, 4]. Wnts, the ligand of *β*-catenin, is secreted by PCa cells and remodels the bone microenvironment, inducing osteoblastic activity and local bone remodeling, which leads to osteosclerotic lesions (also known as osteoblast lesions, which are characterized by increased production of mineralized bone matrix leading to increased bone mineral density) and potentially induces formation of osteolytic lesions (osteoclast-mediated resorption of mineralized bone matrix leading to low bone mineral density). Although destruction of bone by metastatic PCa is the result of dysregulation of both osteoclast and osteoblast activity, osteoblasts remain a key driver of PCa progression in bone [5]. Thus, studying the crosstalk between PCa cells and osteoblasts may help to explain the mechanisms underlying development of PCa in the bone microenvironment.

Recently, increasing evidence has shown that the N6-methyladenosine (m^6^A) modification on RNA plays an important role in cancer development. M^6^A is the most common and conserved RNA modification in mammals, which is written by an m^6^A methyltransferase (such as METTL3/14/16, RBM15/15B, VIRMA, KIAA1492, ZC3H3, and others), is read by m^6^A-binding proteins (such as YTHDF1/2/3, YTHDC1/2, HNRNPA2B1 and IGF2BP1/2/3, among others), and is finally removed by demethylases (such as ALKBH5 and FTO). RNA m^6^A modification can occur in different types of RNA, including mRNA, tRNA, rRNA, circRNA, miRNA and lncRNA, and it plays an important role in regulating RNA alternative splicing, translation, translocation, stability, as well as other processes [6–8]. Most m^6^A sites exist near stop codons and are included in the conserved motif DRACH (D = G/A/U, R = G/A, H = A/U/C) [9].

It has been shown that m^6^A regulation also plays an important role in the development of PCa. For example, the most aggressive PCa is accompanied by elevated total m^6^A levels possibly due to the loss of DNA copy number of two m^6^A “erasers” (FTO and ALKBH5), as well as high expression of m^6^A-related “Reader” and “Writer” genes [10]. Notably, the total level of m^6^A is increased with the progression of PCa [11]; in particular, it is associated with a high Gleason score [10]. This highlights the potential involvement of m^6^A regulation in the development of PCa.

We previously found that a cold-stress response protein, RNA binding motif 3 (RBM3), attenuates the stem-like properties of PCa cells partly by interfering with alternative splicing of CD44 [12]. RBM3 has been shown to be involved in the regulation of various cellular functions, especially cell adaptation and neuroprotection under low temperature environments [13, 14]. Interestingly, as an RNA-binding protein (RBP), RBM3 has also been predicted as a potential m^6^A-related RBP [15]. In the present study, we found that RBM3 is associated with m^6^A regulation in PCa, and that high expression of RBM3 reduces catenin beta 1 (CTNNB1) mRNA stability by upregulating the m^6^A modification in the 3’UTR of CTNNB1, resulting in decreased protein levels of *β*-catenin, downregulation of Wnt signaling, and greatly attenuated stemness remodeling of PCa cells by osteoblasts.

## Results

### RBM3 is negatively correlated with Wnt signaling in bone metastatic PCa

**Fig. 1:**
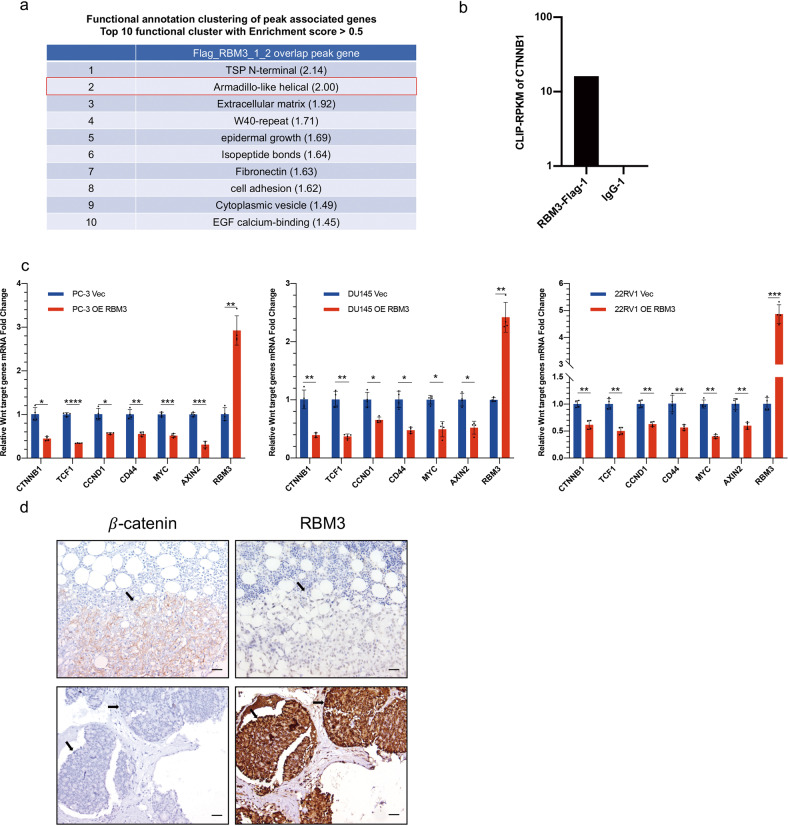
RBM3 is negatively correlates with Wnt signaling in bone metastatic prostate cancer. **a** CLIP-seq cluster analysis shows that RBM3 was closely related to Armadillo helical sequences. **b** Reads Per Kilobase per Million mapped reads (RPKM) of CTNNB1 in CLIP-seq shows that RBM3 could bind to CTNNB1. **c** RT-qPCR analysis of the mRNA expression of *β*-catenin and its downstream target genes in three prostate cancer cell lines. Data are mean ± SD (*n* = 4); *P < 0.05. *P* values were calculated using two-sided paired Student’s *t* test. **d** Representative images of immunohistochemical staining of *β*-catenin and RBM3 in paraffin-embedded human bone metastatic prostate cancer tissue. Black arrows indicate cancer areas; scale bar, 100 µm.

We have previously found that RBM3, a stress response RNA binding protein, attenuates the stem-like properties of PCa cells partly by interfering with CD44 variant splicing [12]. To further explore the function of RBM3 in PCa, we performed CLIP-seq analysis on PC-3 cells overexpressing RBM3-Flag. The functional clustering analysis of genes related to RBM3 binding peaks showed that Armadillo (ARM)-like helical sequence enrichment scores ranked in the top 2 clusters (Fig. 1a). Proteins containing ARM-like helical sequences are a large family, and the classic ARM repeat-containing protein is *β*-catenin which plays an important role in cell growth and development [16]. And the results of the CLIP-seq showed that RBM3 might physically bind to the mRNA of CTNNB1 (Fig. 1b). This raises the possibility that RBM3 may regulate Wnt/*β*-catenin signaling by interfering with CTNNB1 mRNA. In support, overexpression of RBM3 extrinsically not only decreased the mRNA level of CTNNB1, but also significantly inhibited the activation of downstream target genes of *β*-catenin, such as AXIN2, MYC, and others (Fig. 1c).

To further explore the potential relationship between RBM3 and Wnt/*β*-catenin signaling, the protein expression of RBM3 and *β*-catenin was detected by immunohistochemistry in histopathological specimens of PCa bone metastasis. Interestingly, although it was only observed within a very low number of samples, a likely negative association was observed between the expression pattern of *β*-catenin and RBM3, namely, when RBM3 was strongly positive in both the nucleus and cytoplasm and was not limited within the nucleus, the level of *β*-catenin protein was very low (Fig. 1d). On the other hand, when *β*-catenin protein was strongly expressed on both the cell membrane and in the cytoplasm and was not limited to the cell membrane, the expression of RBM3 was relatively weak and limited to the nucleus. Given that translocation of RBM3 from nucleus to cytoplasm, as well as translocation of *β*-catenin from cell membrane to cytoplasm indicate activation of both proteins, this result further supports a potential functional association between RBM3 and Wnt/*β*-catenin signaling.

We have previously found that the mRNA level of RBM3 was decreased in metastatic PCa compared with organ-confined cancer [12], and that protein expression of RBM3 was also decreased in metastatic PCa [17, 18]. As it has been widely accepted that the bone microenvironment endows tumor cells with the capacity for stemness remodeling and secondary metastasis [4], it is reasonable to speculate that the loss of RBM3 expression, which likely favors the activation of Wnt/*β*-catenin signaling, may play a role in stemness remodeling in bone metastases of PCa.

### RBM3 attenuates stem-like properties of PCa cells co-cultured with osteoblasts by inhibiting Wnt/*β*-catenin activation

**Fig. 2:**
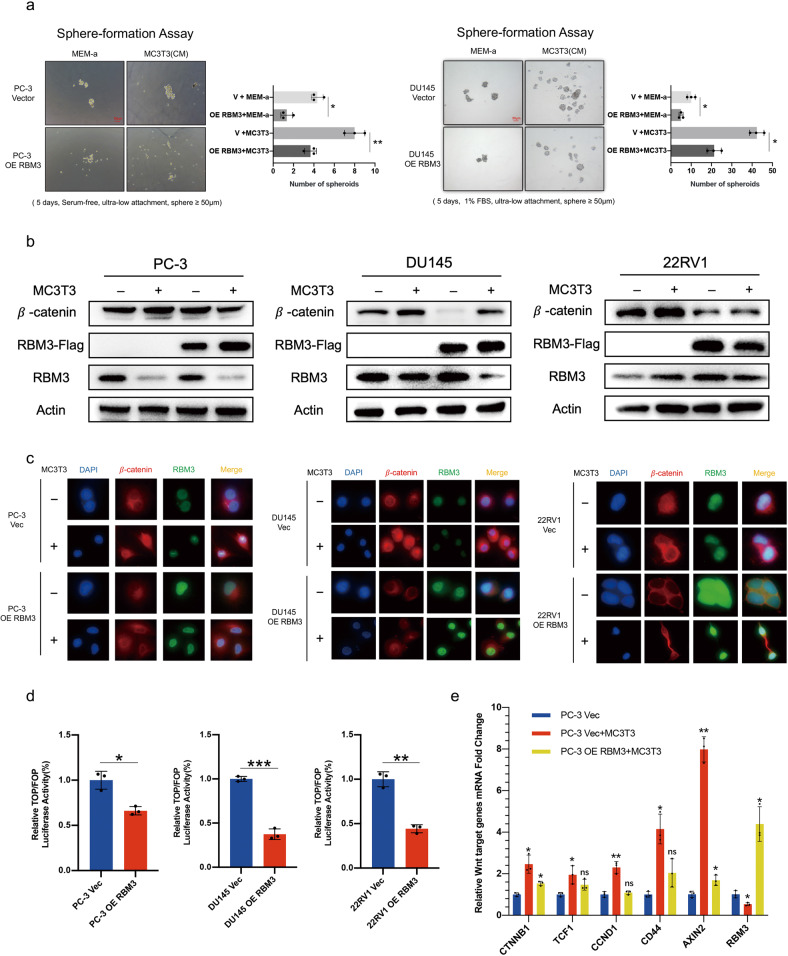
RBM3 attenuates stem-like properties of PCa cells co-cultured with osteoblasts by inhibiting Wnt/*β*-catenin activation. **a** Left column, representative image of prostate cancer cell spheroidization in MEM-a medium; right column, representative image of prostate cancer cell spheroidization in osteoblast-conditioned medium; Among them, PC-3 was cultured under serum-free condition, and DU145 was cultured under 1% FBS condition; scale bar, 50 µm; Data are mean ± SD (*n* = 3); *P < 0.05, **P < 0.01. *P* values were calculated using two-sided paired Student’s t test. **b** The protein expression of *β*-catenin, RBM3, and RBM3-Flag were detected by Western Blot in three prostate cancer cells with or without osteoblast co-culture, and the actin levels were used as the internal controls. Semi-quantitative analysis of the bands was shown in Supplementary 1a. **c** Immunofluorescence staining of the protein expression and subcellular localization of *β*-catenin and RBM3 of three prostate cancer cells with or without osteoblast co-culture. And the most representative parts were cut out for display. See Supplementary Material for original image (Supplementary 1c, d, e); cells were counterstained with 4’, 6-diamidino-2-phenylindole (DAPI) to reveal nuclei. **d** Dual luciferase reporter assay of the Wnt signaling activity in three prostate cancer cells. Data are mean ± SD (*n* = 3); *P < 0.05, **P < 0.01, ***P < 0.001; *P* values were calculated using two-sided paired Student’s *t* test; Renilla fluorescence values were used as the internal controls. **e** RT-qPCR analysis of the mRNA expression of *β*-catenin and its downstream target genes in PC-3 cells with or without osteoblast co-culture. Data are mean ± SD (*n* = 3); *P < 0.05, **P < 0.01. *P* values were calculated using two-sided paired Student’s *t* test.

To test the above hypothesis regarding stemness remodeling in metastatic PCa, we conducted cell sphere-formation assays using PCa cells overexpressing RBM3 or cells bearing empty vector in the presence or absence of serum-free osteoblast conditioned medium (CM). However, DU145 cells could not survive in the serum-free and ultralow-attachment environment so, for these cells, medium containing 1% FBS was used, while 22RV1 cells could not form cell spheres in either serum-free or 1% FBS condition medium. After 5 days of culture, we found that PC-3 and DU145 cells transfected with empty vector and treated with osteoblast CM could form larger and more numerous cell spheroids than cells cultured with normal MEM-*β* medium, indicating that the stem-like properties of these PCa cells were increased when treated with osteoblast CM. On the other hand, the cells overexpressing RBM3 formed fewer and smaller cell spheres compared with cells bearing empty vector even when cultured with osteoblast CM, indicating that RBM3 could inhibit the effect of osteoblast CM on the enhancement of PCa stemness (Fig. 2a). Based on western blot assays, we found that after co-culture with osteoblasts, the total protein levels of *β*-catenin in the three PCa cell lines were increased (Fig. 2b). Immunofluorescence and Western Blot showed that the abundance of *β*-catenin was increased in the nucleus of PC-3 and DU145 cells after co-culture with osteoblasts, while in 22RV1 cells it showed perinuclear aggregation (Fig. 2c; Supplementary 1b). Although *β*-catenin did not show obvious nuclear entry in 22RV1 cells, the total amount of *β*-catenin protein was significantly up-regulated after co-culture. We believe that the different responses of 22RV1 cells compared to co-culture of PC-3 and DU145 cells with osteoblasts may be related to the intrinsic properties of the three cell lines [3, 19], while overexpression of RBM3 inhibited the nuclear and total protein levels of *β*-catenin in PCa cells after co-culture. Interestingly, it was found that the protein levels of endogenous RBM3 were significantly down-regulated in PC-3 and DU145 cells but not in 22RV1 cells after co-culture with osteoblasts. Given that the stemness evidenced by the cell-sphere-formation ability of PC-3 and DU145 but not of 22RV1 cells was likely increased by co-culture with osteoblasts, we believe that downregulation of RBM3 may be important to permit the development of stemness in PCa cells. This result is consistent with our previous study showing overexpression of RBM3 suppressed cell stemness in PCa [19]. In conjunction with our finding of low expression of RBM3 in human pathological specimens of bone metastatic PCa, these results suggest that the bone environment may promote cell stemness of PCa by suppressing the biological function of RBM3.

To verify the effect of RBM3 on Wnt signaling, the reporter system TOPflash was used to detect the transcriptional activity of TCF/LEF that is induced by Wnt/β-catenin signaling. As a negative control, FOPflash mutated at the binding site of the TOPflash sequence was used. As shown in Fig. 2d, RBM3 overexpression in PC-3, DU145, and 22RV1 cells significantly inhibited activation of Wnt signaling in the TOPflash/FOPflash reporter system. In further support of this finding, the mRNA expression of Wnt/*β*-catenin target genes was increased in PC-3 cells after co-culture with osteoblasts, while RBM3 overexpression in PC-3 cells inhibited the activation of Wnt/*β*-catenin downstream genes triggered by the co-culture environment (Fig. 2e). Taken together, these results suggest that RBM3 attenuates cell stemness of PCa in mimic bone microenvironment.

### RBM3 reduces CTNNB1 mRNA stability by upregulating m^6^A methylation

**Fig. 3:**
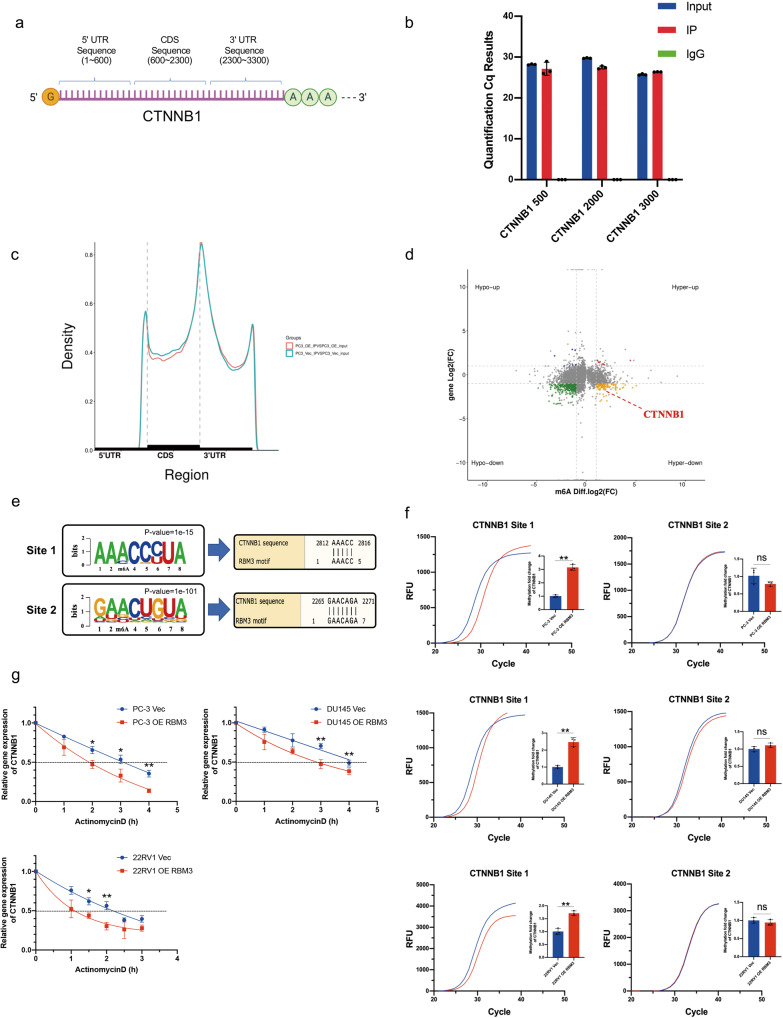
RBM3 reduces CTNNB1 stability by upregulating m^6^A methylation. **a** Divide CTNNB1 into 5’UTR, 3’UTR and CDS sequence. **b** Use IP-grade RBM3 antibody to pull down PC-3 overexpressing RBM3 to obtain RNA which interacts with RBM3. The Input group was diluted to the same concentration as the IP group (about 6 times), and then the amplified Cq value of the three primers of CTNNB1 in the pulled down RNA samples was detected by RT-qPCR. Data are mean ± SD (*n* = 3). **c** RNA region peak density; the light blue curve is the PC-3 Vec group; the red curve is the PC-3 OE RBM3 group; (**d**) Four-quadrant map of RBM3 m^6^A-seq, the abscissa axis is the relative level of m^6^A modification, and the ordinate is the relative level of RNA; after taking log2 of the data in the figure, PC-3 OE RBM3 VS PC-3 Vec is used for display; compared with the control group, CTNNB1 m^6^A level was up-regulated and mRNA level was down-regulated in RBM3 overexpression group. **e** The CTNNB1 m^6^A site corresponding to the RBM3 motif; Site1 is located in the 3’UTR region of CTNNB1, and Site2 is located in the CDS region. **f** Detection of m^6^A modification levels of CTNNB1 Site1 and Site2 in three prostate cancer cells by using the SELECT m^6^A site identification detection system. The curve in the figure is the amplification curve, and the histogram is the fold change of m^6^A modification level calculated by 2-ΔΔCT. Data are mean ± SD (*n* = 3); **P < 0.01. *P* values were calculated using two-sided paired Student’s *t* test. **g** The control and overexpression groups of three prostate cancer cells were treated with actinomycin D (10 ug/mL), RNA samples were collected at different time periods, and the relative RNA levels of CTNNB1 were detected by RT-qPCR. ACTB level was used as the internal controls; Data are mean ± SD (*n* = 3); *P < 0.05, **P < 0.01. *P* values were calculated using two-sided paired Student’s *t* test.

Next, we asked how RBM3 regulates *β*-catenin expression in PCa cells. Given that RBM3 is an RNA-binding protein, and that CLIP-seq showed that RBM3 may bind to CTNNB1 mRNA, a RIP-qPCR was conducted to test whether RBM3 in fact bound to CTNNB1 mRNA. Primers used in RIP-qPCR were designed specifically for different segments of CTNNB1 mRNA, namely, the 5’UTR sequence, 3’UTR sequence and CDS sequence (Fig. 3a). As shown in Fig. 3b, every segment of CTNNB1 mRNA was highly detected after IP with RBM3 antibody. This result suggests that RBM3 truly binds to the mRNA of CTNNB1.

The next question is what happens after RBM3 binding to the mRNA of CTNNB1. There have been some reports indicating the relationship between RBP and m^6^A regulation [20–23]. Interestingly, RBM3 has been predicted to be a potential m^6^A-related RBP [15]. In this regard, an MeRIP-seq experiment was performed on PC-3 cells overexpressing RBM3. The results showed that RBM3 was more likely to bind to the 3’UTR of RNA (Fig. 3c), and the four-quadrant diagram showed that the levels of m^6^A methylation on many RNAs was affected by RBM3 overexpression (Fig. 3d). Importantly, the mRNA level of CTNNB1 was as expected down-regulated by RBM3 overexpression, while, at the same time, the m^6^A level on the mRNA of CTNNB1 was up-regulated (Fig. 3d). This result raises the possibility that RBM3 may affect the m^6^A methylation after binding the mRNA of CTNNB1. To verify this, we first screened the m^6^A canonical motif “DRACH” in the RBM3-binding motifs from the above MeRIP-seq result and found four candidate motifs, two of which could be identified in the mRNA sequence of CTNNB1: one is located in 3’UTR region (Site 1), another is located in CDS region (Site 2; Fig. 3e). Next, we tested if m^6^A methylation was present at these two sites on the mRNA of CTNNB1 following RBM3 overexpression. For this purpose, the SELECT assay was conducted. SELECT detection is a single-base extension and ligation qPCR amplification technology, where if there is methylation modification at the detected site, the amplification efficiency will be reduced. Using this method, we measured the single-based m^6^A level of either Site 1 or Site 2 in CTNNB1 mRNA. The results showed that the m^6^A level at motif Site 1 but not Site 2 was significantly increased in RBM3-overexpressing PCa cells compared with control cells (Fig. 3f). This strongly suggests that RBM3 induces m^6^A methylation at the m^6^A canonical motif site located in the 3’UTR of CTNNB1 mRNA.

Finally, to check the effect of RBM3-induced m^6^A methylation of the 3’UTR of CTNNB1 mRNA on expression of the gene, mRNA stability was examined in PCa cells. After gene transcription was inhibited by actinomycin D treatment, it was found that the decay rate of CTNNB1 mRNA was significantly faster in RBM3-overexpressing cells compared with the control cells, indicating that the m^6^A methylation induced by RBM3 could lead to low stability of CTNNB1 mRNA, resulting in decreased expression of *β*-catenin following RBM3 overexpression (Fig. 3g).

### RBM3 upregulates m^6^A methylation of CTNNB1 mRNA via METTL3

**Fig. 4:**
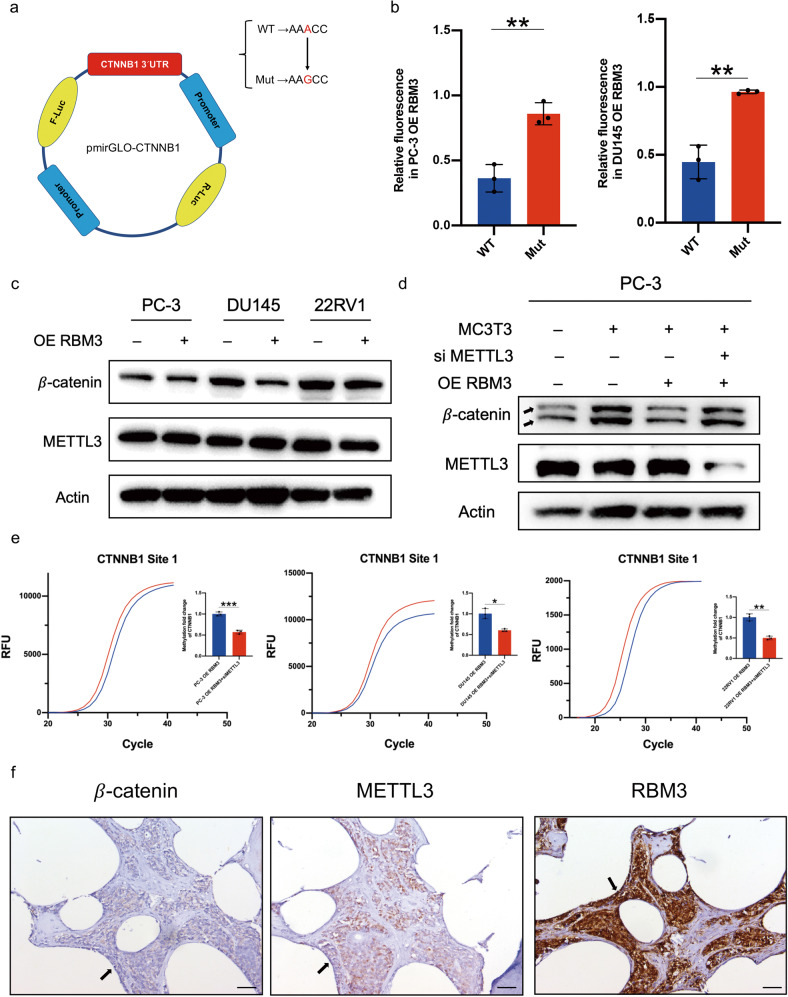
RBM3 upregulates m^6^A methylation of CTNNB1 mRNA via METTL3. **a** The third “A” of the CTNNB1 Site1 was mutated to “G”, and then the wild-type and mutant CTNNB1 Site1 were inserted into the dual-luciferase plasmid. **b** Dual-luciferase plasmids of CTNNB1 Site1 WT or Mut sequence were transferred into prostate cancer cells of the RBM3 overexpressing group, and the relative fluorescence intensity was detected by dual luciferase reporter system; Renilla fluorescence values were used as internal controls. Data are mean ± SD (*n* = 3); **P < 0.01. *P* values were calculated using two-sided paired Student’s *t* test. **c** Western blot was used to detect the protein expression of METTL3 in three prostate cancer cells after overexpression of RBM3, and actin levels were used as the internal controls. Semi-quantitative analysis of the bands was shown in Supplementary 2a. **d** Western blot was used to detect the protein expression of *β*-catenin in PC-3 after co-culture with MC3T3, overexpressing RBM3 or knockdown of METTL3. And actin levels were used as the internal controls. Semi-quantitative analysis of the bands was shown in Supplementary 2b. Indicated band were *β*-catenin main bands with molecular weights of about 90 kDa. **e** SELECT m^6^A site identification detection system was used to detect the m^6^A modification level of CTNNB1 Site1 in three prostate cancer cells after METTL3 knockdown. The curve in the figure is the amplification curve, and the histogram is the fold change of m^6^A modification level calculated by 2-ΔΔCT. Data are mean ± SD (*n* = 3); *P < 0.05, **P < 0.01, ***P < 0.001. *P* values were calculated using two-sided paired Student’s *t* test. **f** Representative images of immunohistochemical staining for RBM3, *β*-catenin, and METTL3 in bone metastasis prostate cancer tissue. Black arrows indicate cancer areas; scale bar, 50 µm.

We next investigated how RBM3 modifies the m^6^A methylation of CTNNB1 mRNA. According to the previous finding that m^6^A methylation of CTNNB1 mRNA on Site 1 decreased mRNA stability, we constructed a reporting system for mRNA stability, in which we inserted the wild type (WT) or mutated (Mut) sequence corresponding to Site 1 of the CTNNB1 mRNA into a dual-luciferase plasmid (Fig. 4a). After introducing this system into PC-3 or DU145 cells overexpressing RBM3, we found that the relative fluorescence intensity in cells bearing the WT sequence of Site 1 was much lower than in cells bearing the Mut sequence, indicating it was highly likely that RBM3 affected the stability of CTNNB1 mRNA by modifying the sequence of Site 1 (Fig. 4b).

We next asked how RBM3 wrote methylation on Site 1 of CTNNB1 mRNA. It has been shown that METTL3 is the major catalytic methyltransferase modifying RNA m^6^A [24]. To test whether METTL3 was involved in this process, protein expression of METTL3 in PCa cells was first examined, which showed that METTL3 protein was expressed in all three PCa cell lines, although overexpression of RBM3 had little effect on METTL3 expression (Fig. 4c). Next, the expression of METTL3 was suppressed by siRNA in PC-3 cells overexpressing RBM3, and then these cells were co-cultured with MC3T3 cells to induce the expression of *β*-catenin. Notably, on one hand, it was consistently shown that the increased expression of *β*-catenin in PC-3 cells induced by co-culturing was decreased by RBM3 overexpression; on the other hand, knocking-down the expression of METTL3 restored the expression of *β*-catenin that had been suppressed by RBM3 overexpression (Fig. 4d), indicating that the impact of RBM3 overexpression on the expression of *β*-catenin required the involvement of METTL3. In support of this, the m^6^A methylation level on Site 1 of CTNNB1 mRNA was also significantly reduced by knocking-down the expression of METTL3 (Fig. 4e), suggesting that METTL3 potentially contributed to the methylation modification of CTNNB1 mRNA on Site 1 induced by RBM3 overexpression.

The potential relationships among the expression of RBM3, *β*-catenin and METTL3 was also observed in human PCa tissue samples, in which RBM3 was strongly stained when *β*-catenin was weakly expressed, whereas METTL3 was always strongly expressed (Fig. 4f), supporting the involvement of METTL3 in the regulation of *β*-catenin by RBM3.

### RBM3 inhibits osteoblast-induced Wnt/*β*-catenin activation in PCa cells by inducing CTNNB1 m^6^A methylation

**Fig. 5:**
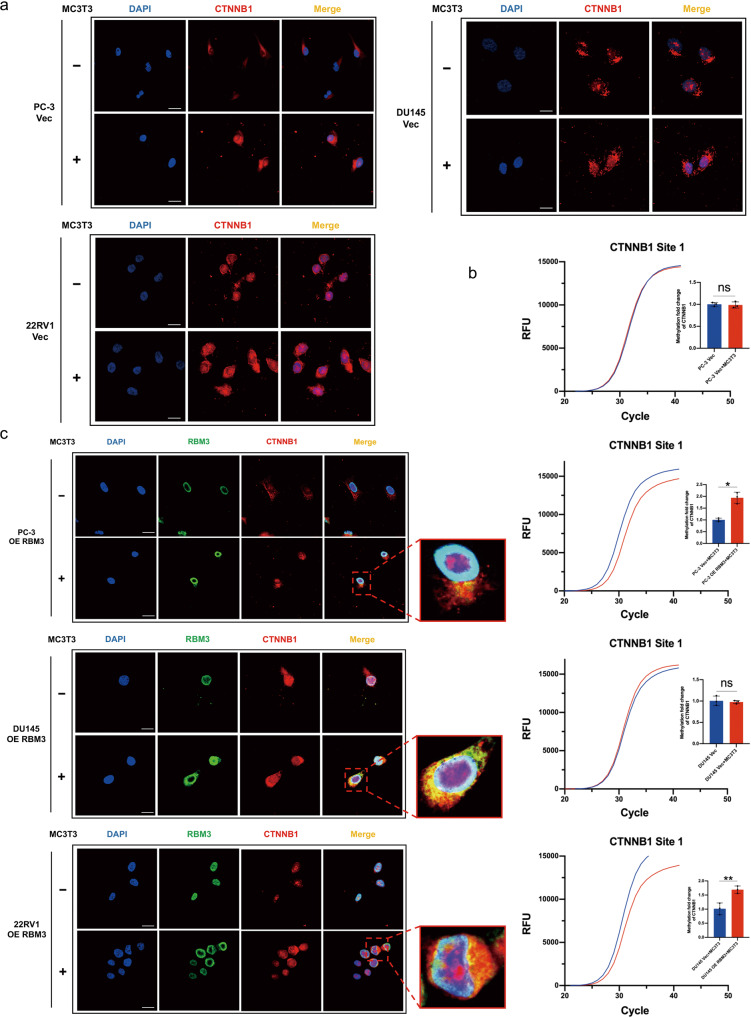
RBM3 inhibits osteoblast-induced Wnt/β-catenin activation in PCa cells by inducing CTNNB1 m^6^A methylation. **a** FISH staining of the expression and subcellular localization of CTNNB1 mRNA in three prostate cancer cells with or without osteoblast co-culture. Cells were counterstained with 4’, 6-diamidino-2-phenylindole (DAPI) to show nuclei; scale bar, 20 µm. **b** SELECT m^6^A site identification detection system was used to detect the m^6^A modification level of CTNNB1 Site1 in prostate cancer cells with or without osteoblast co-culture. The curve in the figure is the amplification curve, and the histogram is the fold change of m^6^A modification level calculated by 2-ΔΔCT. Data are mean ± SD (*n* = 3); *P < 0.05, **P < 0.01. *P* values were calculated using two-sided paired Student’s *t* test. **c** FISH and IF co-staining of the expression and subcellular localization of CTNNB1 mRNA and RBM3 protein in RBM3 overexpressing PCa cells with or without osteoblast co-culture. Cells were counterstained with 4’, 6-diamidino-2-phenylindole (DAPI) to show nuclei; scale bar, 20 µm.

To determine whether RBM3 truly modified m^6^A methylation of the mRNA of CTNNB1 in PCa cells in the mimicked bone microenvironment, we used fluorescent in situ hybridization (FISH) as well as detection of single-based m^6^A level. Firstly, it was found that the mRNA abundance of CTNNB1 in PCa cells was significantly increased after co-culture with osteoblast cells, accompanied by extranuclear aggregation of CTNNB1 mRNA (Fig. 5a); in contrast, the m^6^A level on the mRNA of CTNNB1 at Site 1 did not change in this process (Fig. 5b). However, when co-cultured PCa cells overexpressed RBM3, m^6^A methylation on the mRNA of CTNNB1 at Site 1 was significantly increased compared to co-cultured PCa cells without RBM3 overexpression (Fig. 5b). Furthermore, the FISH study showed that, in those RBM3 overexpressing PCa cells, the RBM3 protein tended to aggregate perinuclearly from the nucleus and co-localize there with CTNNB1 mRNA when co-cultured with osteoblast cells (Fig. 5c). Thus, in our mimic bone microenvironment system, we observed co-location of RBM3 protein with CTNNB1 mRNA perinuclearly, together with m^6^A methylation increasing simultaneously on the mRNA of CTNNB1. These results further support that RBM3 may inhibit osteoblast-induced Wnt/*β*-catenin activation in PCa cells by inducing CTNNB1 m^6^A methylation, which in turn reduces mRNA stability as well as the expression level of *β*-catenin.

### The protein expression of RBM3 is decreased while the mRNA expression of CTNNB1 is increased in PCa cells xenografted in a mouse bone microenvironment

**Fig. 6:**
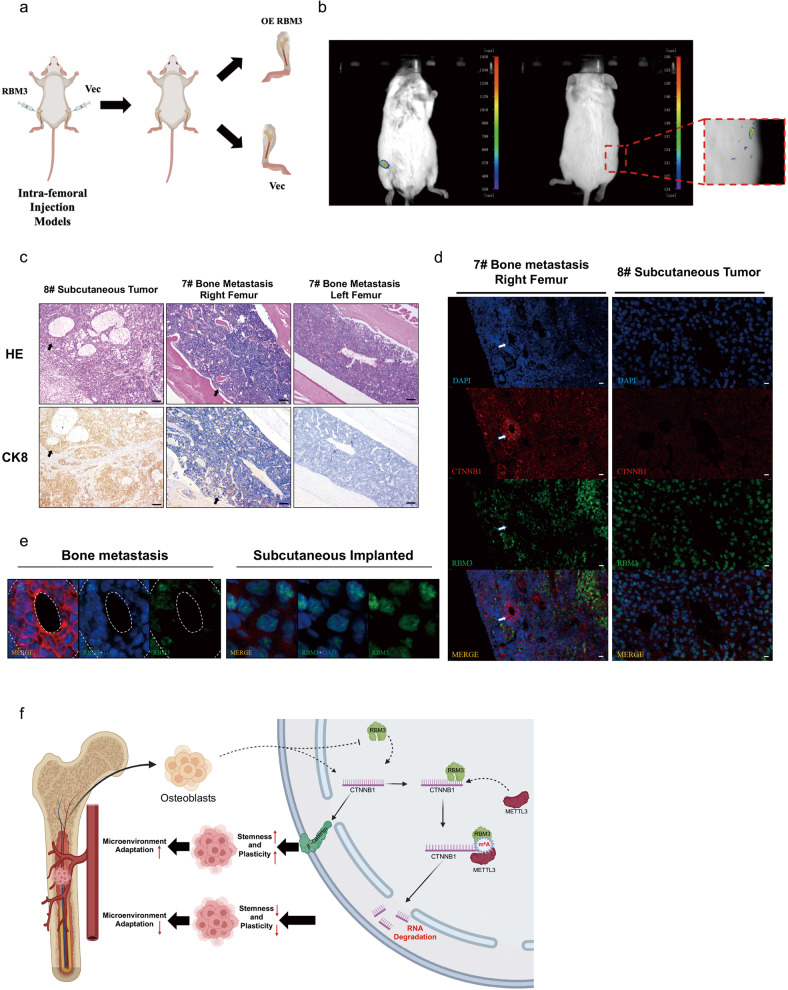
The protein expression of RBM3 is decreased while the mRNA expression of CTNNB1 is increased in PCa cells xenografted in a mouse bone microenvironment. **a** The intramedullary injection of mouse femur simulates bone metastasis of prostate cancer. Prostate cancer cells were injected into the bone marrow cavity of mouse femurs by using 29 G syringe, RBM3 overexpressing PCa cells were injected into the left femur, and Vector PCa cells were injected into the right femur. The number of cells injected into each femur was 5 × 10^5. **b** In vivo imaging of mouse with intramedullary injection of PC-3 cells. The left side is the supine position image, and the right side is the prone position image. In both postures, obvious fluorescence signals can be found in the right hindlimb. The average fluorescence intensity in mouse model was shown in supplementary 3a. **c** Representative images of immunohistochemical staining of CK8 and HE is staining in 7# bone metastasis model and 8# subcutaneous implanted tumor; scale bar, 100 µm. **d** FISH and IF co-staining of the expression and subcellular localization of CTNNB1 mRNA and RBM3 protein in left leg of 7# bone metastasis model and 8# subcutaneous implanted. Cells were counterstained with 4’, 6-diamidino-2-phenylindole (DAPI) to show nuclei; scale bar, 10 µm. **e** Representative images of tumor areas in 7# bone metastasis model and 8# subcutaneous implanted tumor. **f** Proposed model for RBM3 regulation of CTNNB1 in the bone microenvironment. Osteoblasts upregulated the RNA abundance of CTNNB1 in tumor cells. RBM3 functions as a stress-responsive protein in a novel tumor microenvironment. RBM3 protein binds to CTNNB1 mRNA, and then METTL3 writes m^6^A methylation modification. This process ultimately leads to the decrease of CTNNB1 stability as well as *β*-catenin protein expression, which reduces the stemness and plasticity of tumor cells. Thus, reducing the adaptability of tumor cells in the bone microenvironment.

To better understand the effect of RBM3 on tumor growth in the metastatic microenvironment, PC-3 cells bearing a luciferase gene with or without RBM3 overexpression were injected into the bone marrow cavity of mouse femurs (see Supplementary 3 for the specific experimental process) as well as subcutaneously. To reduce the individual differences, PC-3 cells overexpressing or not expressing RBM3 were injected into either side of the leg in the same mouse (Fig. 6a). After 7 weeks, there was an obvious fluorescence signal detected in the right hindlimb of the mice, where PC-3 cells without RBM3 overexpression were injected, but no fluorescence signal was detected in the left hindlimb, where PC-3 cells with RBM3 overexpression were injected (Fig. 6b). Similarly, only PC-3 cells without RBM3 overexpression formed xenograft tumors subcutaneously in mice. These results agreed well with our previous finding that RBM3 overexpression strongly inhibited xenograft tumor growth in vivo [12].

Furthermore, immunohistochemical staining confirmed that CK8-positive PCa cells were present in the xenografted tumors in the bone or under the skin (Fig. 6c). Next, a fluorescence in situ hybridization assay for CTNNB1 was performed along with immunofluorescence staining for RBM3 in xenografted tumors in bone as well as in subcutaneous tumors (Fig. 6d). Accordingly, as compared with subcutaneous tumor cells, CTNNB1 mRNA was significantly up-regulated while RBM3 protein was significantly down-regulated in cancer cells growing in bone (Fig. 6e), which was consistent with our in vitro observations (see Result 2). This result suggests that the activation of *β*-catenin signaling may be important for cancer survival in the bone microenvironment, while lack of RBM3 may be necessary for this process to occur.

According to these in vitro and in vivo results, we hypothesized that overexpression of RBM3 reduced the mRNA stability of CTNNB1 by affecting its m^6^A methylation and decreased the expression of *β*-catenin. This in turn inhibited the stemness remodeling in PCa cells by the bone microenvironment, which consequently impeded the adaptive survival of the tumor cells (Fig. 6f).

## Discussion

It is well accepted that the “stress” faced by cancer cells in the tumor microenvironment is an important driving force that affects tumor progression [25–28]. Similarly, cancer cells metastasized to the bone microenvironment also face various stresses, such as metabolic stress, inflammation, shear stress, and others [29–32]. In this scenario, the response of cancer cells mediated largely by a group of stress-response proteins like RBM3 plays a crucial role in deciding the fate of cancer cells in the tumor microenvironment; that is “to be or not to be”. In addition, the adaptive stress-response regulation of cancer cells is not only related to cell survival in the new environment, but also leads to the remodeling of the cancer cells and their evolution into a more aggressive phenotype, for example, the stemness feature, which helps cancer cells develop drug-resistance or secondary dissemination [33–37]. During the stress-response, the RNA modification, as a rapid and effective regulatory mechanism, may become the key method by which tumor cells adapt to the change in the microenvironment [4, 38–41]. As a stress-response protein, RBM3 also has RNA-binding properties which play a crucial role in determining the fate of cancer cells in the tumor microenvironment [42–44]. We have previously shown that the expression of RBM3 was significantly decreased in cells that survived stressful environments, such as high temperature, chemotherapy conditions or a soft agar environment, favoring the enhanced stemness feature of surviving cells by inducing cell quiescence [12, 45, 46]. In the present study, we similarly observed that interaction of PCa cells with the bone microenvironment reduced the expression of RBM3, and this potentially “permitted” the activation of Wnt *β*-catenin signaling which is critically important for bone metastasis of PCa. Thus, the present study may extend our understanding of the inhibitory role of RBM3, particularly in bone metastasis of PCa.

Activation or loss of *β*-catenin signaling may play different roles in different stages of the development of PCa [47], with the greatest impact on promoting stem cell-like properties and the self-renewal ability of tumor cells [48–50]. The present study showed that RBM3 negatively regulates the protein expression of *β*-catenin and inhibits the activation of *β*-catenin signaling, especially under co-culture with osteoblasts. In agreement with our previous study [12], we found that, whether in vivo or in vitro, the inhibitory effect of RBM3 overexpression on the stemness characteristics of PCa cells was prominent, suggesting a role for RBM3 as stemness suppressor gene in PCa. Indeed, the protein level of RBM3 has been shown to be significantly downregulated in bone metastases compared with orthotopic tumors [17, 18].

There is accumulating evidence indicating that m^6^A methylation is closely involved in the development of many cancers including PCa, especially metastatic PCa [10, 51–54]. As a highly effective RNA modification, m^6^A methylation and its relationship with RBPs has been primarily studied in cancers [20, 55–58]. Even more so, RBP-targeted therapies aiming at RNA regulation are also under research [59–61]. For example, Zhao et al. reported that the RNA-binding protein SORBS2 binds to the 3’ UTR of WFDC1 or IL-17D and enhances its mRNA stability, then further inhibits the metastasis of ovarian cancer [62]. In the present study, we observed the colocalization of RBM3 protein with the mRNA of CTNNB1 when cancer cells were co-cultured with osteoblast cells to mimic a bone microenvironment. Importantly, it was verified that RBM3 indeed directly binds with the mRNA of CTNNB1 and affects the m^6^A methylation at a particular site; this in turn reduces the stability of mRNA and results in a decreased level of *β*-catenin. As an RNA binding protein, RBM3 has been shown to function as an RNA chaperone to affect RNA stability [12, 63–65]; it is, however, the first time, to the best of our knowledge, that RBM3 has been shown to impact RNA stability by modulating m^6^A methylation at particular site in mRNA. This epigenetic-based regulatory approach is very different from conventional protein-based modulation of *β*-catenin. Interestingly, this epigenetic-based regulatory approach may exist in the metastatic microenvironment and contribute to the dynamic modulation of Wnt/*β*-catenin signaling in supplementary to the conventional regulation pathway. In another aspect, Feng et al. found that circ-CTNNB1 promotes overall m^6^A levels by interacting with RBM15 (m^6^A-associated RBPs) and thus leads to osteosarcoma progression [66], providing another example of the relationship between RBP and m^6^A methylation in mRNA. In this context, the present study indicates a novel role for RBM3 as an m^6^A-associated RBP.

Recently, a growing number of studies have shown that the expression of CTNNB1 mRNA can be regulated by m^6^A modification. For instance, Xu et al. reported that, as an m^6^A-related “reader”, YTHDF3 enhanced the stemness profile of melanoma by promoting the translation of CTNNB1 [67]. Liu et al. found that when METTL3 was knocked down in hepatoma cells, m^6^A methylation level as well as the stability of CTNNB1 mRNA was reduced [68]. Interestingly, in one study, Li et al. demonstrated that m^6^A modification at the 5’UTR of CTNNB1 mRNA in Hela cells affects RNA stability [69]. By contrast, in our study, we found that the m^6^A methylation induced by RBM3 located at the 3’UTR of CTNNB1 mRNA was critical for maintaining RNA stability. The different effects of m^6^A methylation at different site on the stability of CTNNB1 mRNA is intriguing and should be further studied.

In conclusion, in the present study, we found that high expression of RBM3 in PCa cells promotes m^6^A methylation of CTNNB1 to reduce its RNA stability and decrease the expression of *β*-catenin, and thus may impede stemness remodeling of PCA cells induced by the osteogenic microenvironment. Thus, restoring the expression of RBM3 that is suppressed in the bone microenvironment or modulating the m^6^A methylation of CTNNB1 may be important therapeutic approaches for inhibiting bone metastasis of PCa.

## Methods

### Cell lines and cell co-culture

All cell lines were obtained from the cell bank of Shanghai Institutes for Biological Sciences, Chinese Academy of Sciences. All cell lines were identified by cell species, STR analysis and mycoplasma detection to ensure no contamination by other cells or foreign microorganisms. Human PCa cell lines PC-3, DU145 and 22RV1 were cultured in DMEM (Gibco, China) containing 10% FBS (Gibco). Mouse embryonic osteoblast precursor cells (MC3T3-E1) were cultured in MEM-a (Gibco) containing 10% FBS (Gibco). All cells were cultured at 37 °C in a humidified environment with 5% CO_2_. PCa cells and osteoblasts were co-cultured in a 6-well plate with 0.4 µm Transwell co-culture chambers (Corning, USA). PCa cells were plated in the lower layer of the chamber 24 h before co-culture, and then osteoblasts were seeded in the upper layer of the chamber. The ratio of osteoblasts to PCa cells was 5:1. To provide sufficient nutrition, 3 ml of MEM-a with 10% FBS was added to the lower chamber and 1.5 ml of serum-free medium was added to the upper chamber. The co-culture duration was 3 days, and the medium was replaced every 24 h.

### Crosslinking Immunoprecipitation and high-throughput sequencing (CLIP-seq)

PC-3 cells were UV irradiated (CL-3000, UVP, Germany) once for 400 mJ/cm2, and lysed in ice-cold wash buffer. Cells lysis was performed in cold Wash buffer (1 × PBS, 0.1% SDS, 0.5% NP-40 and 0.5% sodium deoxycholate) supplemented with a 200 U/mL RNase inhibitor (2313U, Takara, Japan) and protease inhibitor cocktail (B14001,bimake, China) and incubate on ice for 30 min. Clear cell lysate by centrifugation at 10,000 rpm for 10 min at 4 °C. Add RQ I(1 U/μl, M610 A, Promega, Madison, WI, USA) to a final concentration of 0.05 U/μl and incubate in a water bath for 40 min at 37 °C. Immediately afterward, a stop solution was added to the lysates to quench DNase. The mixture was then vibrated vigorously and centrifuged at 13,000 x g at 4 °C for 20 min to remove cell debris. Then RNA digestion by MNase (2910 A, Thermo Scientific, USA) was performed. For immunoprecipitation, the supernatant was incubated overnight at 4 °C with 5 μg Flag-antibody (80010-1-RR, Proteintech, China). The immunoprecipitants were further incubated with protein A/G Dynabeads (26162, Thermo Scientific, China) for 2 h at 4 °C. After applying to magnet and removing the supernatants, the beads were sequentially washed with lysis buffer, high-salt buffer (250 mM Tris 7.4, 750 mM NaCl, 10 mM EDTA, 0.1% SDS, 0.5% NP-40 and 0.5 deoxycholate), and PNK buffer (50 mM Tris, 20 mM EGTA and 0.5% NP-40) for two times, respectively. Resuspend the beads in of Elution buffer (50 nM Tris 8.0, 10 mM EDTA and 1% SDS). Incubate the suspension for 20 min in a heat block at 70 °C to release the immunoprecipitated RBP with crosslinked RNA and vortex. Remove the magnetic beads on the separator and transfer the supernatant to a clean 1.5 ml microfuge tube. After protein gel electrophoresis, a region 75 kDa (~220 nt of RNA) above the protein size is excised and proteinase K (B14001, bimake, China) treated to isolate RNA. The RNA was purified with Trizol reagent (15596-018, Ambion, USA). The data was analyzed with assistance by Appreciate The Beauty Of Life (Wuhan, China).

### RNA extraction and real-time quantitative PCR

Total RNA was extracted using a total RNA extraction kit (Vazyme, China) according to the manufacturer’s instructions. cDNA was synthesized using HiScript III All-in-one RT SuperMix (Vazyme). Real-time quantitative PCR was performed using ChamQ SYBR Color qPCR Master Mix (Vazyme), and ACTB was used as an internal control gene. The real-time PCR reaction was prepared as follows: 2.5 μL ddH2O, 5 μL 2 × SYBR mix, 0.5 μL forward primer, 0.5 μL reverse primer, mixed with 2 μL of cDNA template. PCR reactions included initial denaturation at 95 °C for 30 s, followed by 40 cycles of denaturation at 95 °C for 5 s and annealing at 60 °C for 30 s. Relative expression was calculated by the ΔΔCT method.

### Immunohistochemistry

Bone metastatic samples were derived from six patients with PCa, as well as from mouse models. The paraffin-embedded tissue sections were obtained from the Pathology Department of Liaoning Cancer Hospital Affiliated to Dalian University of Technology. The screening criteria for bone metastasis patients were PSA > 100 ng/mL, metastatic pathological tissue sections were positive for prostate cancer specific antigen and determined by professional pathologists. As all metastatic specimens were derived from orthopedic surgery without knowing primary prostate cancer, the Gleason scores for these primary tumors are lack. This study complies with the Declaration of Helsinki and was approved by the Institutional Ethics Committee. Written informed consent was obtained from all patients and the samples used were tested while maintaining anonymity. Tissue samples were fixed with 10% neutral buffered formalin and embedded in paraffin. When performing immunohistochemistry, sections were deparaffinized and boiled in citrate solution for 35 min. After 10 min incubation with 3% hydrogen peroxide, sections were blocked with 5% BSA for 1 h at room temperature, and then incubated with primary antibodies (Abcam catalog: ab134946, USA; CST catalog: 9562, 86132, USA) at 4 °C overnight. Afterwards, sections were incubated with the appropriate secondary antibodies (Gene Tech, China) and then stained with DAB and hematoxylin.

### Lentiviral infection, plasmids and siRNA construction

The RBM3 plasmid was purchased from Genechem (Shanghai, China). The vector-bearing RBM3 construct or empty vector was transfected into 293 T cells by calcium phosphate transfection method with 2nd Generation Packaging System Mix (Abmgood, Canada). The viral supernatant was collected within 48 h and filtered through a 0.45 µm filter (Merck Millipore, Germany). Lentivirus and 8 μg/mL polybrene (Sigma-Aldrich, USA) were added to the PCa cells for infection. After 72 h, 2 μg/ml puromycin (Invivogen, USA) was added to cells for 1 week to select cell clones stably expressing RBM3 or empty vector. Selected cell clones were cultured in medium containing 200 ng/mL of puromycin to maintain RBM3 overexpression. CTNNB1 3’UTR WT and Mut dual fluorescein plasmids were commissioned by BGI (Shenzhen, China), and siRNAs targeting RBM3 or METTL3 were commissioned by RiboBio Sciences (Guangzhou, China).

### Ultra-low attachment sphere-formation assay

PCa cells were seeded at 500 cells per well into an ultra-low adhesion 24-well plate and cultured with 500 μL of FBS-free MEM. The cell spheroids were measured and counted under a microscope after 5 days.

### Western blot

The cell samples were washed twice with cold PBS and then lysed by RIPA lysis buffer (Beyotime, China). The protein supernatant was collected by centrifugation and the protein concentration was measured using a BCA protein assay kit (Beyotime). Proteins were heated at 95 °C for 5 min, and 20 μg protein was loaded into each lane and separated by 10% SDS-PAGE in Tris-glycine running buffer, then transferred to PVDF membranes (Millipore). Membranes were blocked with TBST containing 5% skim milk for 1 h at room temperature and ere then incubated with primary antibodies overnight at 4 °C. After washing three times with TBST 5 min per wash, the membranes were incubated with HRP-conjugated secondary antibody (Zsbio catalog: ZB-2306, China) in TBST containing 5% skim milk for 1 h. Membranes were then washed three more times with TBST and visualized using Pierce ECL Plus (Thermo Fisher, USA).

### Immunofluorescence

The PCa cells were seeded in a 12-well plate and treated under various conditions for 3 days. After washing with PBS, the cells were fixed with 4% paraformaldehyde for 15 min, and permeabilized with 0.3% Triton X-100 (Beyotime) for 20 min. After washing with PBS, the cells were blocked with 2% BSA for 1 h, incubated with primary antibody (Abcam, USA; CST, USA) overnight at 4 °C, and then incubated with fluorescent secondary antibody (Jackson, USA) for 1 h. Finally, the cells were treated with anti-quenching DAPI (Beyotime) and observed under a fluorescence microscope.

### TOP/FOP flash assay

The sequences of 8xTOPFlash-miniP and 8xFOPFlash-miniP were obtained from (https://www.addgene.org) and the plasmids were constructed assisted by Genechem. 8xTOPFlash-miniP and 8xFOPFlash-miniP were transfected into PCa cells using the calcium phosphate transfection method. The firefly luciferase values and Renilla luciferase values were detected by a dual-luciferase reporter gene detection kit (Vazyme) according to the manufacturer’s instructions. First, the relative fluorescence intensity of firefly fluorescein and Renilla fluorescein in the TOP and FOP groups were compared to obtain the relative fluorescence intensity, and then the relative fluorescence intensity of the TOP group was compared with the FOP group.

### RNA immunoprecipitation (RIP)

RIP-qPCR was performed by using the Magna RIP™ RNA-Binding Protein Immunoprecipitation Kit (Millipore, USA). RIP lysis buffer was used to lyse cells according to the manufacturer’s instructions. Cell extracts were incubated with magnetic beads conjugated with anti-Argonaut 2 (AGO2; Millipore) or control anti-immunoglobulin G (IgG) antibodies. Cell extracts were incubated for 6 h at 4 °C. After protein beads were removed, qRT-PCR was performed to detect target RNAs.

### M^6^A immunoprecipitation and m^6^A sequence analysis

The m^6^A immunoprecipitation was performed according to well-established procedures [70]. Total RNA was isolated and purified using TRIzol reagent (Invitrogen, USA) following the manufacturer’s procedure. The RNA amount and purity of each sample was quantified using NanoDrop ND-1000 (NanoDrop, USA). Poly (A) RNA is purified from 30 μg total RNA using Dynabeads Oligo (dT)25-61005 (Thermo Fisher, USA) using two rounds of purification. Then the poly(A) RNA was fragmented into small pieces using Magnesium RNA Fragmentation Module (NEB, cat.e6150, USA) under 86 °C 7 min. Then the cleaved RNA fragments were incubated for 2 h at 4 °C with m^6^A-specific antibody (Synaptic Systems, cat.202003, Germany) in IP buffer (50 mM Tris-HCl, 750 mM NaCl and 0.5% Igepal CA-630). Then the IP RNA was reverse-transcribed to create the cDNA by SuperScript™ II Reverse Transcriptase (Invitrogen, cat.1896649, USA), which were next used to synthesise U-labeled second-stranded DNAs with E. coli DNA polymerase I (NEB, cat.m0209, USA), RNase H (NEB, cat.m0297, USA) and dUTP Solution (Thermo Fisher, cat.R0133, USA).An A-base is then added to the blunt ends of each strand, preparing them for ligation to the indexed adapters. Each adapter contains a T-base overhang for ligating the adapter to the A-tailed fragmented DNA. Single- or dual-index adapters are ligated to the fragments, and size selection was performed with AMPureXP beads. After the heat-labile UDG enzyme (NEB, cat.m0280, USA) treatment of the U-labeled second-stranded DNAs, the ligated products are amplified with PCR by the following conditions: initial denaturation at 95 °C for 3 min; 8 cycles of denaturation at 98 °C for 15 sec, annealing at 60 °C for 15 sec, and extension at 72 °C for 30 sec; and then final extension at 72 °C for 5 min. The average insert size for the final cDNA library was 300 ± 50 bp. At last, we performed the 2 × 150 bp paired-end sequencing (PE150) on an illumina Novaseq™ 6000 (LC-Bio Technology Co., Ltd., Hangzhou, China).

### NSELECT m^6^A site identification

The single site m^6^A methylation of CTNNB1 was detected by the SELECT m^6^A site identification kit (Epbiotek, China). First, the relative ratio of CTNNB1 RNA levels between the treatment group and the control group was obtained according to real-time quantitative PCR, and an equivalent amount of CTNNB1 RNA was used for reverse transcription. Then, the single-base ligation was performed by using SELECT DNA polymerase and SELECT ligase. Finally, real-time quantitative PCR was conducted using the special primers designed for the CTNNB1 m^6^A site.

### mRNA stability assay

PCa cells were inhibited with 10 μg/mL actinomycin D (Aladdin, China) for 0 h, 1 h, 2 h, 3 h and 4 h, separately. The relative mRNA levels of CTNNB1 at each time point were detected by real-time quantitative PCR and data processing was performed by Graphpad Prism 8 (USA).

### Fluorescence in situ hybridization (FISH) and immunofluorescence

CTNNB1 mRNA probes and the fluorescence in situ hybridization kit were purchased from RiboBio. The subcellular localization of CTNNB1 mRNA in PCa cells was detected by FISH according to the manufacturer’s instructions. In brief, the steps were as follows: [1] Fix with 4% paraformaldehyde for 10 min [2]. Permeabilize with 0.5% TritonX-100 for 5 min [3]. Prepare hybridization solution, hybridization solution I/II/III and pre-hybridization solution [4]. Add the pre-hybridization solution and block at 37 °C for 30 min [5]. Prepare the probe hybridization solution with the 37 °C-hybridization solution and CTNNB1 probe. Mix in set proportions [6]. Add the probe hybridization solution at 37 °C overnight in the dark [7]. Wash with hybridization solution 1/2/3 at 42 °C in sequence. [8] Block with 2% BSA for 1 h and immunofluorescence primary antibody at 4 °C overnight. [9] Incubate with fluorescent secondary antibody for 1 h at room temperature. [10] Add DAPI dropwise to cover the slides. Finally, the slides were photographed using a confocal laser scanning microscope (Leica, Germany).

### Intra-femoral injection mouse model

All animal procedures were approved by the Institutional Animal Care and Use Committee (IACUC) at SPF (Beijing) Biotechnology Co., Ltd., Beijing, China, and all the mice used in the experiment were male at 5–6 weeks old (including 4 NOD mice and 10 nude mice). A total of 5 × 10^5^ PC-3 cells were resuspended in 25 μL PBS and then injected into the femoral platform of the mice with a 29 G syringe. The left leg of the mice was injected with cells overexpressing RBM3, while the right leg was injected with cells bearing empty vector. A detailed description of the animal experimental protocol will be available in the supplementary materials (Supplementary 3). The status of the mice was observed every day. After 7 weeks, fluorescein-D (MCE, USA) was injected intraperitoneally for in vivo imaging. Once the xenografted tumor was detected in the femora of mice, the tumor tissues were harvested for further experiments. All animal experiments were performed according to the National Institutes of Health Guide for the Care and Use of Laboratory Animals and approved by the Animal Care and Use Committees of China Medical University Animal Experiments Ethics Committee.

### Statistical analysis

All in vitro experiments were repeated at least three times. Data are shown as means ± SEM and statistical analysis was performed using SPSS 22.0 (IBM, USA) or Graphpad Prism 8. Two-sided *p*-values <0.05 were considered significant.

## Supplementary information

Supplementary Figure legends

supplementary 1

supplementary 2

supplementary 3

Original images of western blot

Checklist CDDIS-22-4423

## Data Availability

All data generated or analyzed during this study are available from the corresponding author on reasonable request.
