# Supplementary material for: RBM3 suppresses stemness remodeling of prostate cancer in bone microenvironment by modulating N6-methyladenosine on CTNNB1 mRNA
Source: Cell Death Dis. 2023 Feb 7;14(2):91. doi: 10.1038/s41419-023-05627-0 (PMC9905585; doi:10.1038/s41419-023-05627-0)
Supplement: Supplementary file 1 — Supplementary Figure legends [file 41419_2023_5627_MOESM1_ESM.docx]

**Supplementary. 1. A),** Semi-quantifying the intensities of the Western Blot bands by Image J. **B),** The cell nuclear or cytoplasm protein expression of 𝛽-catenin and RBM3 were detected by Western Blot in prostate cancer cells with or without osteoblast co-culture. The actin levels were used as the cytoplasm protein internal controls. The lamin B1 levels were used as the nuclear protein internal controls. **C)**, Immunofluorescence staining of the protein expression and subcellular localization of 𝛽 -catenin and RBM3 of PC-3 cells with or without osteoblast co-culture. Cells were counterstained with 4', 6-diamidino-2-phenylindole (DAPI) to reveal nuclei. **D)**, Immunofluorescence staining of the protein expression and subcellular localization of 𝛽 -catenin and RBM3 of DU145 cells with or without osteoblast co-culture. Cells were counterstained with 4', 6-diamidino-2-phenylindole (DAPI) to reveal nuclei. **E)**, Immunofluorescence staining of the protein expression and subcellular localization of 𝛽 -catenin and RBM3 of 22RV1 cells with or without osteoblast co-culture. Cells were counterstained with 4', 6-diamidino-2-phenylindole (DAPI) to reveal nuclei.

**Supplementary. 2.** **A)**, Semi-quantifying the intensities of the Western Blot bands by Image J. **B)**, Semi-quantifying the intensities of the Western Blot bands by Image J.

**Supplementary. 3.** **A),** The color scale bar depicts the photon flux emitted from the mice, **p* <0.05. Student’s *t* test was used. **B)**, The mouse hind legs were immobilized to show the position of the femur. **C)**, Using the tissue scissors to separate the skin and the patellar ligament was exposed. **D)**, Inserting needle vertically and rotary from the notch above the patellar ligament to penetrate the hard bone, and then reaching the marrow cavity. Continue to insert the needle to reach the hard epiphysis. At this time, the length is about 5/6 of the length of the 29G needle. After withdrawing part of the needle, push the syringe and inject 25µL of PBS (containing tumor cells, with a concentration of 2X10^4/µL). **E)**, After the needle is completely withdrawn, the wound is properly sutured and bandaged.
