# Supplementary figures and images for: RBM3 suppresses stemness remodeling of prostate cancer in bone microenvironment by modulating N6-methyladenosine on CTNNB1 mRNA
Source: Cell Death Dis. 2023 Feb 7;14(2):91. doi: 10.1038/s41419-023-05627-0 (PMC9905585; doi:10.1038/s41419-023-05627-0)

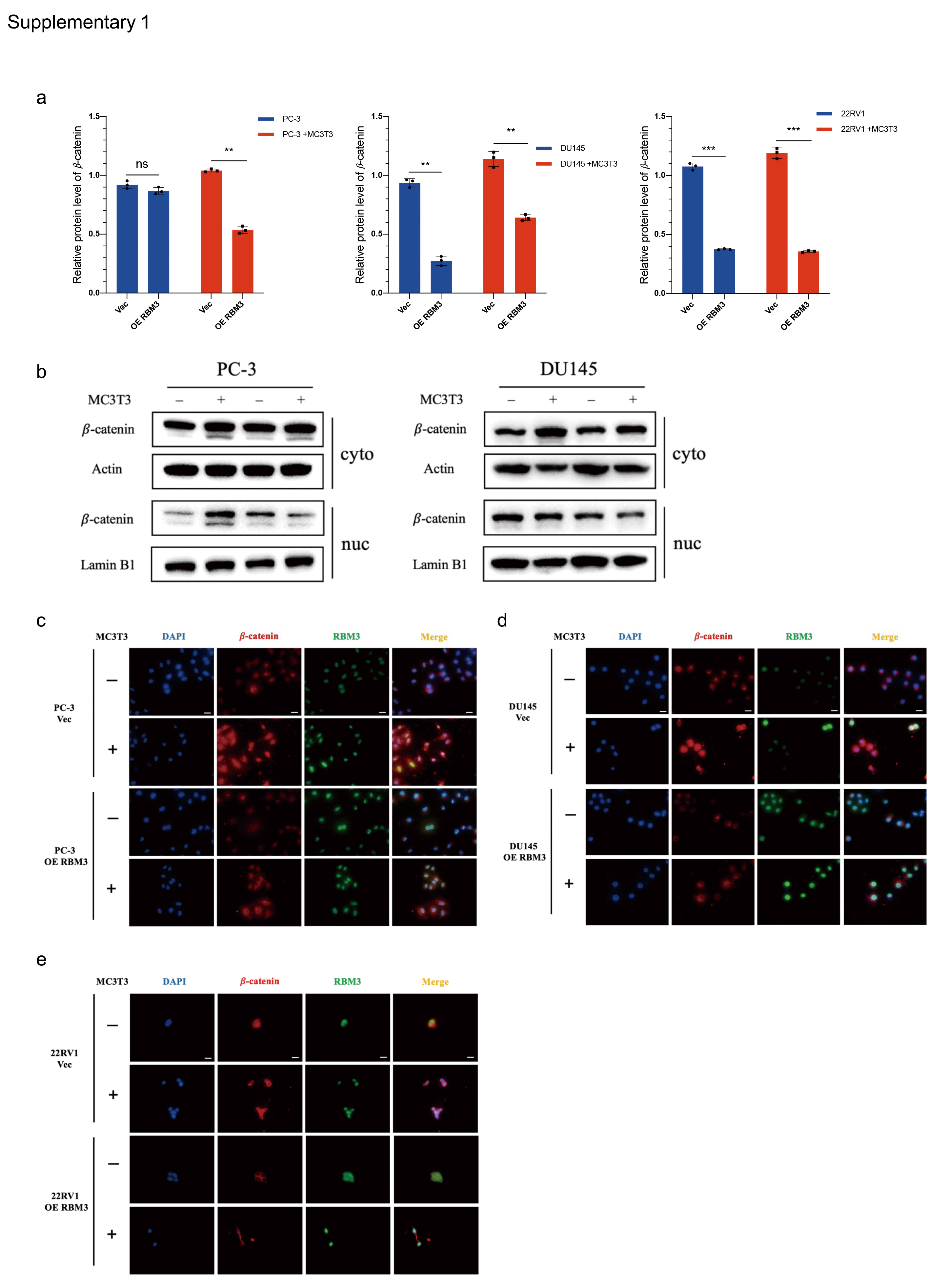

Supplement: Supplementary file 2 — supplementary 1 [file 41419_2023_5627_MOESM2_ESM.jpg]

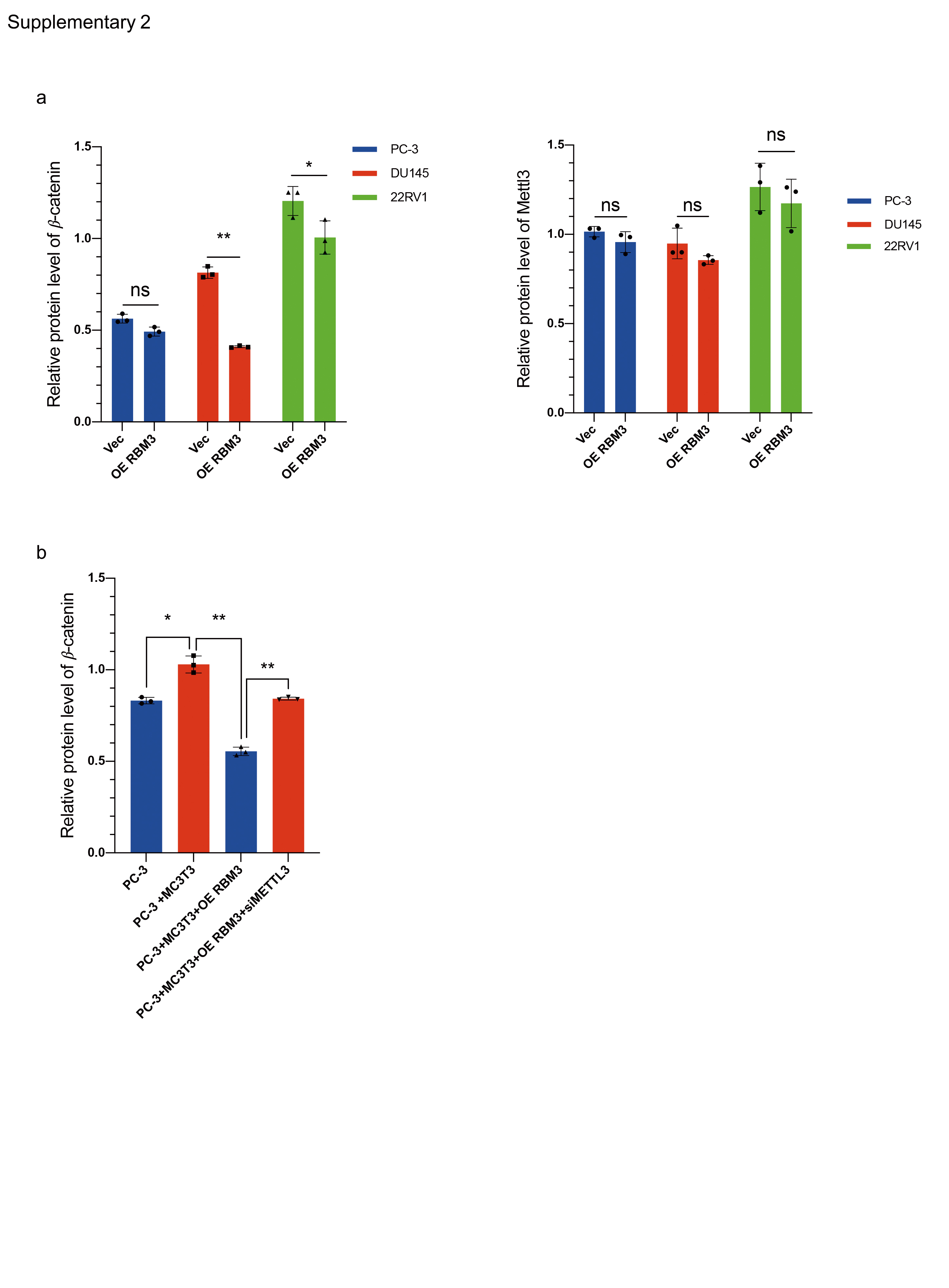

Supplement: Supplementary file 3 — supplementary 2 [file 41419_2023_5627_MOESM3_ESM.jpg]

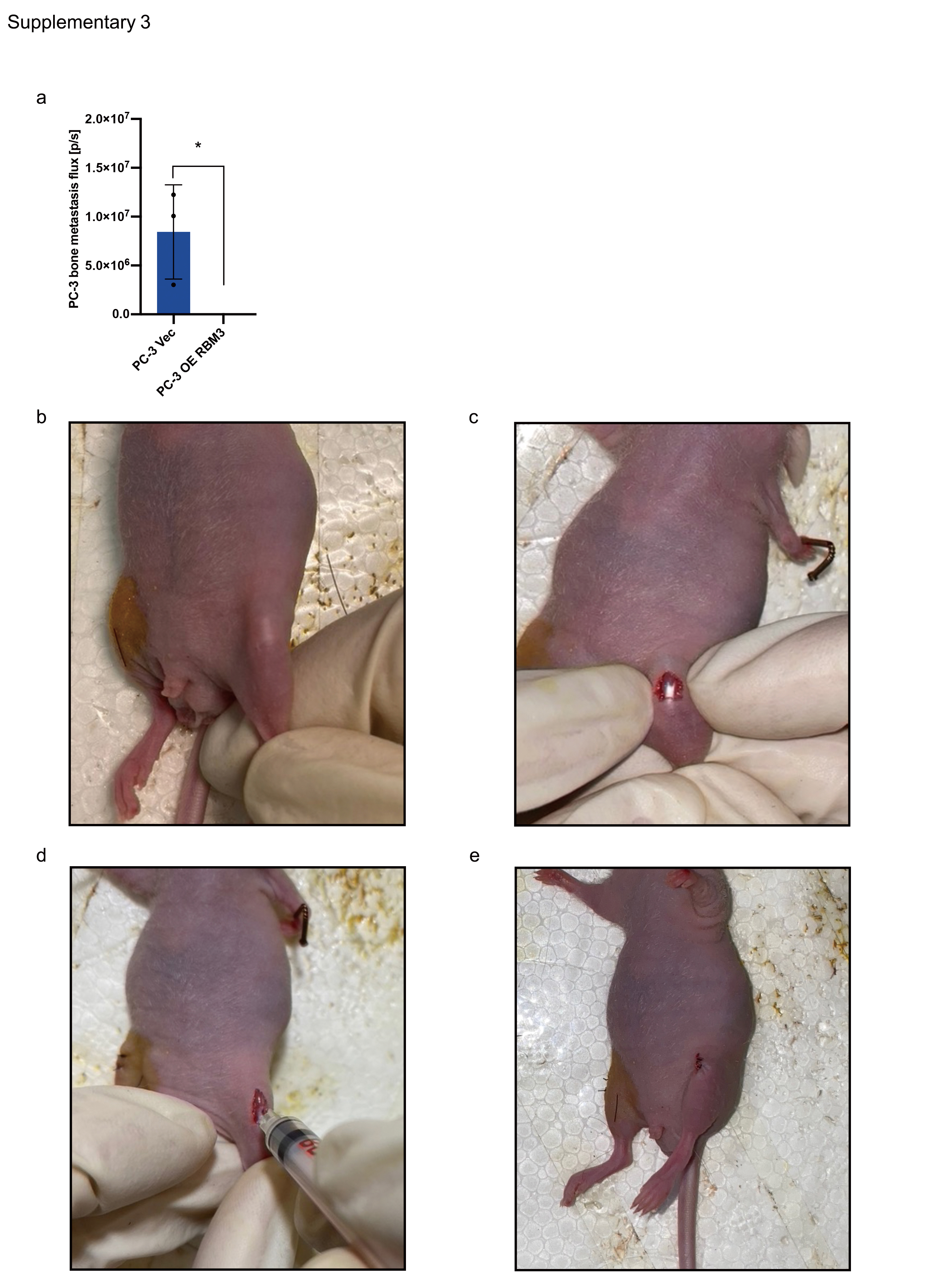

Supplement: Supplementary file 4 — supplementary 3 [file 41419_2023_5627_MOESM4_ESM.jpg]

Figure 2b

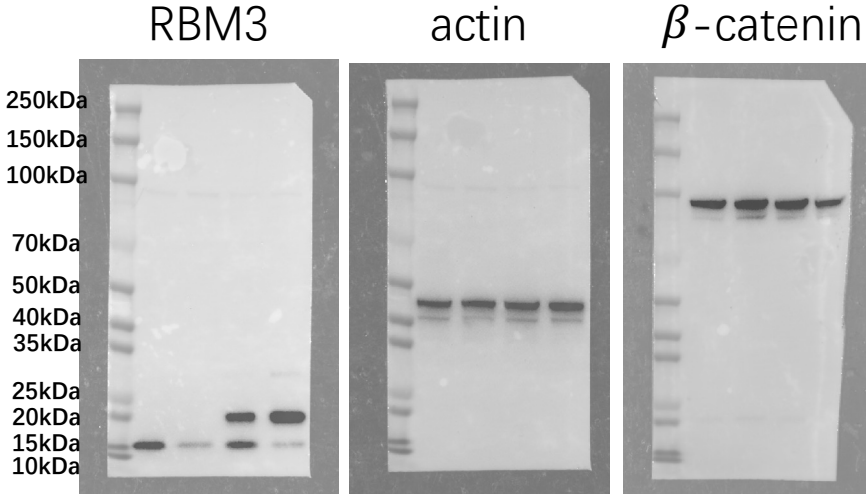

PC-3+MC3T3

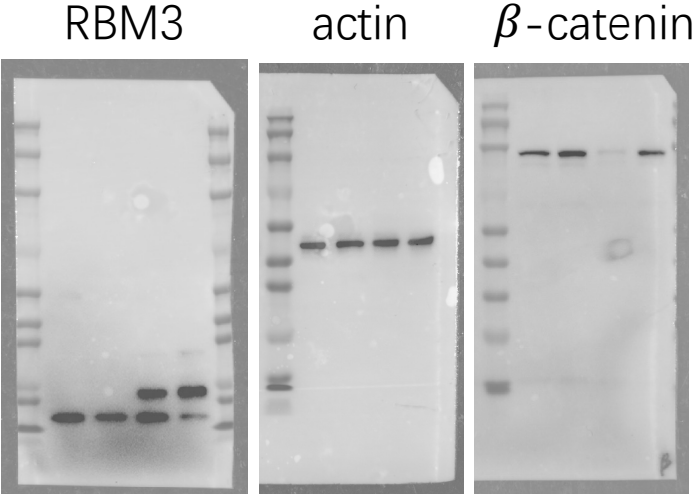

DU145+MC3T3

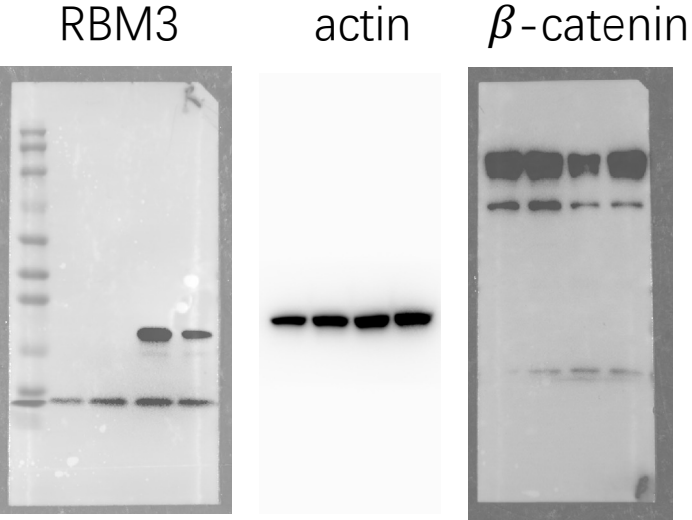

22RV1+MC3T3

Figure 4c

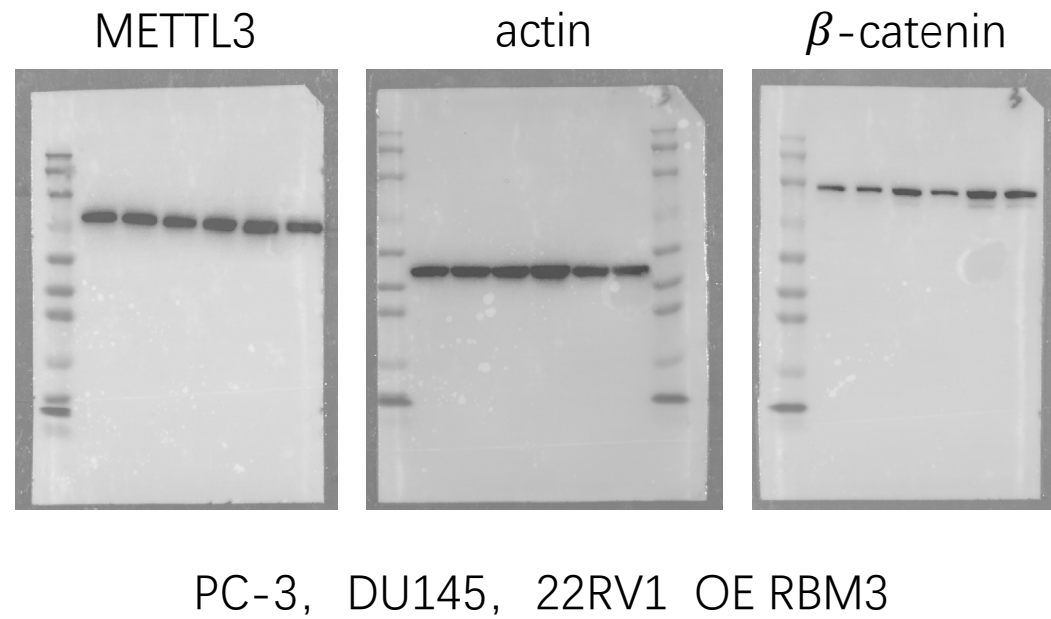

Figure 4d

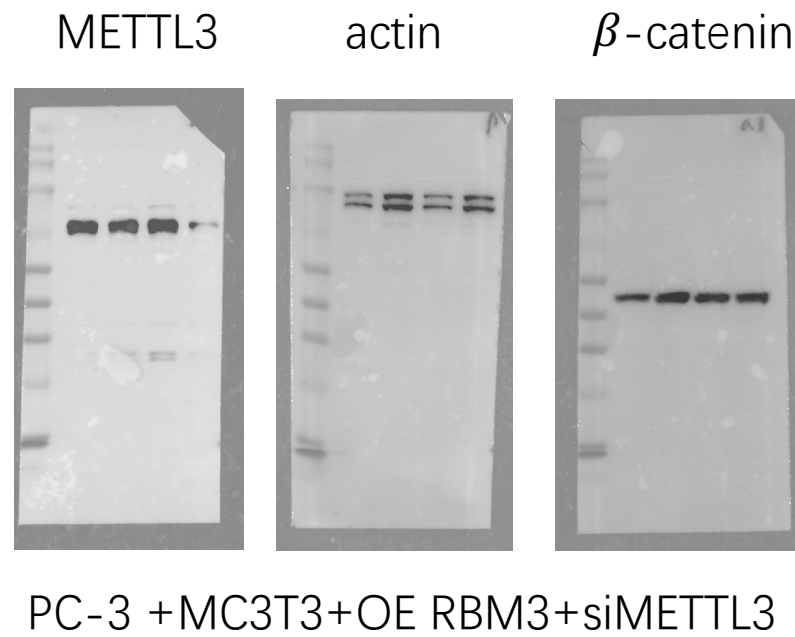

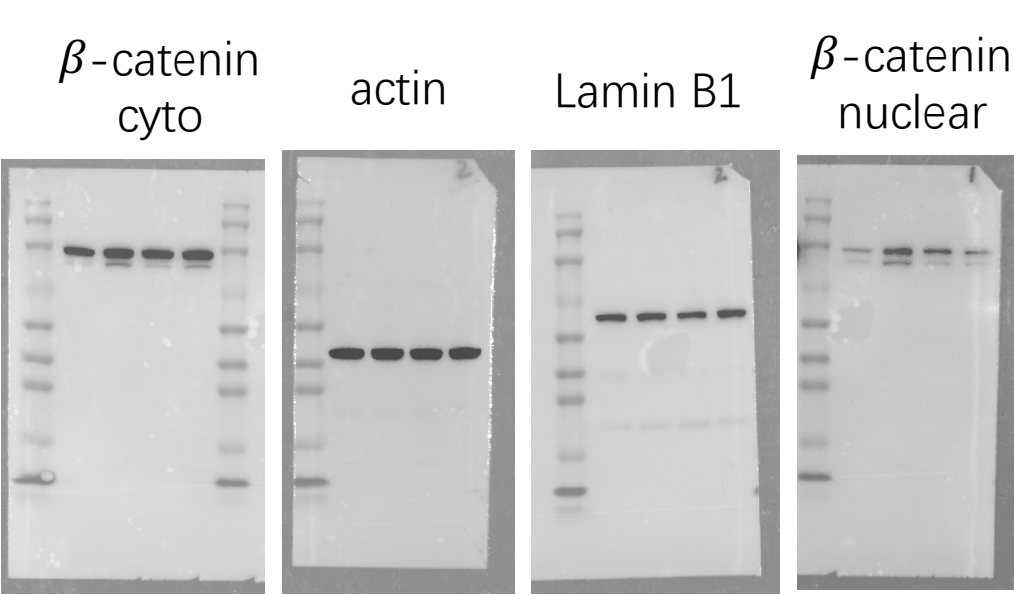

PC-3 +MC3T3

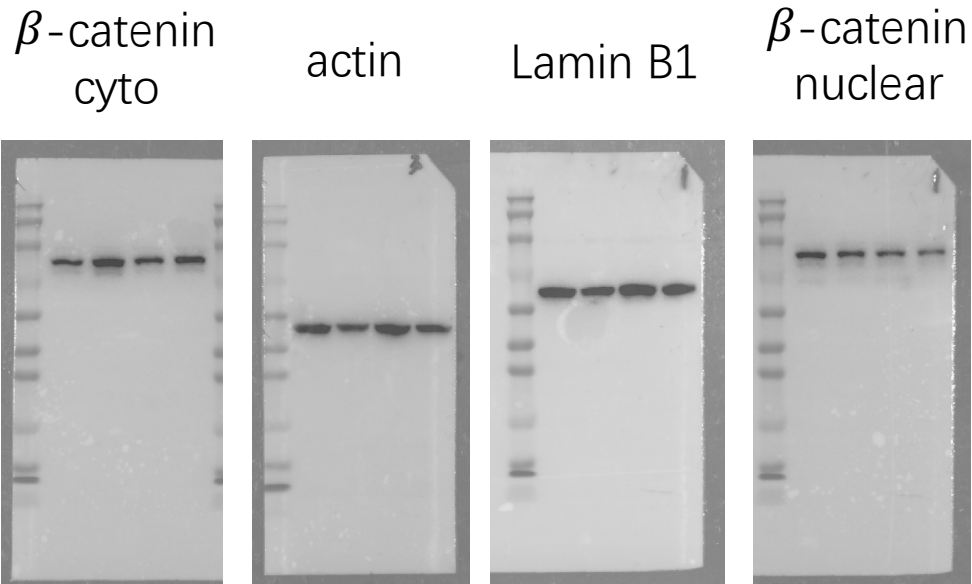

DU145 +MC3T3

Supplement: Supplementary file 5 — Original images of western blot [file 41419_2023_5627_MOESM5_ESM.pdf]
